# *Streptococcus gordonii* septic arthritis : two cases and review of literature

**DOI:** 10.1186/1471-2334-12-215

**Published:** 2012-09-13

**Authors:** Jean cyr Yombi, Leila Belkhir, Sylvie Jonckheere, Dunja Wilmes, Olivier Cornu, Bernard Vandercam, Hector Rodriguez-Villalobos

**Affiliations:** 1 Departement of Internal Medicine and Infectious Diseases, Cliniques Universitaires Saint Luc, Université catholique de louvain, 10 avenue hippocrate, Brussels 1200, Belgium; 2 Departement of Orthopaedic Surgery, Cliniques Universitaires Saint Luc, Université catholique de louvain, 10 avenue hippocrate, Brussels, 1200, Belgium; 3 Departement of Microbiology, Cliniques Universitaires Saint Luc, Université catholique de louvain, 10 avenue hippocrate, Brussels, 1200, Belgium

**Keywords:** Septic arthritis, *Streptococcus gordonii*, Prosthetic joint infection, Endocarditis

## Abstract

**Background:**

Despite advances in antimicrobial and surgical therapy, septic arthritis remains a rheumatologic emergency that can lead to rapid joint destruction and irreversible loss of function. In adults, *Staphylococcus aureus* is the most common microorganism isolated from native joints. *Streptococcus gordonii* is a prominent member of the viridans group of oral bacteria and is among the bacteria most frequently identified as being primary agent of subacute bacterial endocarditis. To the best of our knowledge, *Streptococcus gordonii* has not yet been described as agent of septic arthritis.

**Case Presentation:**

We describe here two cases of septic arthritis due to *Streptococcus gordonii*. It gives us an opportunity to review epidemiology, diagnosis criteria and management of septic arthritis.

**Conclusion:**

Although implication of *S. gordonii* as aetiologic agent of subacute endocarditis is well known, this organism is a rare cause of septic arthritis. In this case, the exclusion of associated endocarditis is warranted.

## Background

Despite advances in antimicrobial and surgical therapies, septic arthritis remains a rheumatologic emergency that can lead to rapid joint destruction and irreversible loss of function. In adults, *Staphylococcus aureus* is the most common microrganism isolated from native joints, found in 40–60% of cases in large series [1]. After *S. aureus*, β-hemolytic streptococci are common cause of bacterial arthritis; in contrast, viridans streptococci are rarely associated with joint infection [2]. *Streptococcus gordonii* is a prominent member of the large category of viridans streptococci of oral bacteria that prevails primarily on tooth surface [3]. *S. gordonii*, among others, is well known for its ability to colonise damaged heart valves and is among the bacteria most frequently identified as being primary aetiological agent of subacute bacterial endocarditis [4]. However, to the best of our knowledge, this species has not yet been described in the literature as agent of septic arthritis (SA). We here describe two cases of septic arthritis due to *Streptococcus gordonii*.

## Case presentation

### Case n°1

A 62-year-old man, born in Turkey, presented to the Orthopaedics Clinic with a painful, erythematous and swollen left knee that had appeared seven days prior to examination. There was no history of fever or chills, no prior history of dental care or any invasive procedure. Past medical history revealed an occlusion of the left central retinal vein (2003), atrial fibrillation, a history of hypertension and hypercholesterolaemia. Current medications included amiodarone, amlodipin and simvastatin. Physical examination revealed poor dental status, cardio-respiratory examination was normal (BP of 130/70 mmHg, HR of 60 bpm), as well as the abdominal examination. Body temperature was checked at 37.5°C. Examination of the left knee confirmed tenderness and erythema associated with a joint effusion. Lab studies disclosed a C-reactive protein (CRP) of 28.8 mg/dL, elevated white blood cell count (WBC) (neutrophil count of 10550 cells/mm^3^). Standard X-Ray of the left knee showed osteoarthritis. Aspiration of the knee revealed purulent fluid with a WBC count of 28800 cells/mm^3^ (90% of neutrophils) and absence of urate crystals. No microorganism was observed on Gram-stain of the synovial fluid. Culture of this liquid yielded alpha-haemolytic streptococci. The organism was identified by mass-spectometry (MALDITOF) and Phoenix system (Becton Dickinson, USA) as *Streptococcus gordonii*. Identification was confirmed by (16S)rRNA gene sequencing. Blood cultures remained negative after 5 days of incubation. Tranoesophageal ultrasound of the heart was normal.

Treatment for septic arthritis was undertaken, and arthroscopic lavage of the joint performed. Microbiology of the synovial biopsy and fluid collected during the procedure also revealed *Streptococcus gordonii*. All isolates showed susceptibility to penicillin, ampicillin, cefotaxime, erythromycin, clindamycin and vancomycin. Antibiotics (ampicillin 2 g 4 hourly) were given intravenously for 2 weeks, and then switched to oral moxifloxacin 400 mg od + rifampicin 600 mg/day for 2 weeks, for total treatment duration of 4 weeks. Follow-up was satisfactory, with a complete clinical and functional recovery.

### Case n° 2

A 78-year-old female presented to the Orthopaedics Clinic with painful swelling of the left knee that had appeared 4 months prior to examination, with marked worsening 4 weeks before admission. She described general malaise and chills as well as a body temperature of 37.7°C. She denied any recent dental care or invasive procedure. Her medical history was marked by degenerative arthritis of the left knee, treated with total knee arthroplasty 9 months prior to presentation. She had a quick and complete recovery after surgery. Her past medical history consisted of Parkinson’s disease treated with levodopa, depression treated with escitalopram, past history of alcohol abuse, and compressive cervical myelopathy which had been treated with surgery. Upon examination, BP was 120/60 mmHg, HR 80 bpm, and temperature 37,7°C. Cardiac examination revealed a systolic murmur (2/6). Examination of the extremities confirmed a warm and tender left knee with moderate effusion. Lab tests disclosed a CRP of 10.25 mg/dL, WBC-count was normal. CT-Scan of the knee reaveled a suspicion of loosening of the prosthesis and enabled a synovial-fluid aspiration. Investigation of the aspirate showed WBC at 16300 cells/mm^3^ (92% of neutrophils). Microscopic examination of Gram-stained synovial fluid was negative for microorganisms. Pure culture of alpha-hemolytic streptococci was recovered after 24 h of incubation of synovial fluid inoculated into aerobic BACTEC system. Organism was identified as viridans group streptococci and later confirmed as *Streptococcus gordonii* by (16S)rRNA gene sequencing (99,9% of identitity). This orgainsm was recovered from other synovial fluid samples by using the same technique, but multiple blood cultures (Bacted system, Becton Dickinson, USA) remained sterile after 15 days of incubation. Transoesophageal echocardiography showed a vegetation on the aortic valve with major aortic regurgitation. Diagnosis of aortic endocarditis was thus confirmed. Gentamycin (5 mg/kg/day) and oral rifampicin (900 mg/day) were thus added to the treatment that had been started after synovial aspiration (iv ampicillin 2 g 4-hourly).

Treatment for chronic post-operative prosthetic joint infection was undertaken, i.e. removal of prosthesis, soft tissue debridement and spacer. Culture of synovial biopsy obtained during surgery yielded pure culture of *S. gordonii*. The patient underwent aortic valve replacement and then, a revision knee arthroplasty was performed in a second operative time. Both surgeries had an uncomplicated follow-up. The total duration of antibiotic (shifted to amoxicilline 3 g/day + rifampicin 900 mg/day) was 12 weeks.

## Discussion

All major surveys on septic arthritis demonstrate that predominant causative organisms of septic arthritis are either staphylococci or streptococci [5-13]. These organisms account for 91% of cases [6]. Gram-negative organisms are more common in older or immunocompromised patients than in young adults. Anaerobic organisms rarely cause septic arthritis, but are more common when there is a history of penetrating trauma [5,14]. The *viridans* group streptococci is however rarely the causative agent of septic arthritis. *S. viridans* group is a heterogenous group of organisms with complex and controversial taxonomy. Today, this group is classified into 6 majors groups: *S. mutans* group, *S. salivarius* group, *S. anginosus* group, *S. mitis* group and *S. bovis* group [15]. Based on phenotypic reactions, the *S. mitis* group can be further divided into *S. sanguinis* (formely known as *S. sanguis*) that includes *S. gordonii,* and *S. mitis.* The viridans group streptococci are part of the commensal flora and have low virulence, and infection with this microorganism usually appears on a previously injured focus [2,15]. However, its association with dental caries and bacterial endocarditis has been well established [2,16]. Comprehensive reviews on the topic does not mention viridans group streptococci as aetiological agents in septic arthritis [5]. However, some isolated cases have been identified, mainly affecting the knee, sternoclavicular, acromioclavicular and sacroiliac joints [2,17-20]. In one of these cases, septic arthritis was associated with bacterial endocarditis [20]. Blankstein et al. [18] described a minor trauma shortly preceding development of septic phenomenon. Alpha-haemolytic streptococci have been implicated in 15 cases of spondylodiscitis, including *S. mitis* group (*S. mitis* and *S. sanguinis*) and *S. anginosus*[21]. Other members of Streptococcus viridans from *S. mitis* group (*S. sanguinis*, and *S. oralis*) have been described in case-reports as agent of septic arthritis [22-24]. We found no case of *Streptococcus gordonii* septic arthritis in modern literature.

Risk factors for development of joint sepsis include: rheumatoid arthritis (RA) or osteoarthritis, prosthetic joints, low socioeconomic status, intravenous drug abuse, alcoholism, diabetes mellitus, previous intra-articular corticosteroid injection and cutaneous ulcers [5].

A prospective community survey of bacterial arthritis found that a number of factors seem to be associated with poor prognosis. These include older age, pre-existing joint disease and the presence of synthetic material within the joint [25].

Septic arthritis typically presents as a hot, swollen, tender joint or joints with a reduced range of movement. Symptoms are usually present for less than 2 weeks at presentation, but significant delay may occur, particularly with low-virulence organisms, tuberculosis and prosthesis infection. Large joints are more commonly reported than small joints. In up to 60% of cases, the affected joints are the hip or the knee [5,8,26,27]. When there is pre-existing inflammatory joint disease, such as RA, symptoms in the affected joint, or joints, are out of proportion to disease activity detected in other joints [5].

Our first patient is an older patient with poor dental status. We postulated that he experienced transitory bacteriaemia (despite the fact that multiple blood cultures upon admission were negative) and that pre-existing severe degenerative arthritis of the knee promoted bacterial graft on that focus. The second patient is also an older woman with pre-existing valvulopathy and knee arthroplasty. We also postulated that she experienced transitory bacteriaemia and developed endocarditis and prosthetic joint infection.

Prompt microscopic analysis and culture of synovial fluid are fundamental diagnostic tools in the evaluation of possible joint sepsis, enabling diagnosis (of both sepsis and crystal-induced arthritis) to be confirmed rapidly. Culture is more sensitive than microscopy alone, as synovial fluid Gram-staining is positive in only 50% of cases.

Cytology performed on synovial fluid shows WBC-count of 100.000 cells/mm^3^ on average (between 25000 and 250000 cells/mm^3^). A cell-count of more than 50.000 cells/mm^3^ with more than 90% of neutrophils makes the diagnosis of septic arthritis highly probable [28]. Coutlakis et al., in their retrospective study on 202 patients, showed that when cell-count on synovial fluid is over 100.000/mm^3^, 77% of patients had septic arthritis, while 47% of patients were diagnosed with SA if cell-count was between 50.000 and 100.000/mm^3^. Below the 50.000/mm^3^-cut-off, diagnosis of SA was less probable [29], though it does not exclude it. A septic arthritis with a WBC-count < 25.000/mm^3^ can be encountered mainly in patients with a history of cancer, those on corticosteroids or in iv-drug abuser, though neutrophil-count remains higher than 90%. In knee arthroplasty, a synovial fluid leucocyte-count of more than 1.7 × 10^3^/mm^3^ had a sensitivity of 94% and a specificity of 88% for prosthetic joint infection (PJI) [30]. Synovial polymorphonuclear neutrophil differential higher than 65% had a sensitivity of 94% and a specificity of 98% for PJI.

Synovial fluid aspiration also enables a search for crystals, to exclude gout or microcrystalline arthritis.

Other laboratory studies can be performed, like glucose levels: (SA : glucose < 40 mg/dL or 50% of glycemia) or lactates (high levels). Though they are not specific, they make diagnosis of septic arthritis more likely [31,32].

The cornerstone of rapid and reliable confirmation of diagnosis of suspected septic arthritis is microbiological examination of synovial fluid. The sensitivity for detection of a causative organisms is, however, often disappointing. It is generally accepted that samples of freshly aspirated joint fluid should be sent to the laboratory for immediate analysis. Different factors have been implicated in the failure to detect the causative organism from patients with septic arthritis including small volume of cultured specimen, purulent exudates that could play an inhibitory effect on bacterial grownth and lower concentration of bacteria in synovial fluid. In this matter, inoculation of synovial fluid into blood culture medium such as BACTEC system (Becton Dickinson diagnostic instrument systems) or paediatric isolator tubes enhances recovery of microorganisms. Inoculation of sterile samples such as synovial fluid directly into BACTEC system is a usual practice in our institution and yielded the aetiologic agent in the two above-mentionned cases. Identification of viridans group streptococci remains difficult by currently manual or automated methods. This, together with taxonomic problems in this group of organisms, have made identification at the species level not always accurate and frequently absent in many reported cases of septic arthritis by viridans group streptococci. Matrix-associated laser desorption ionization-time of fligtht (MALDITOF) is a rapid and cost-effective tool for identification of bacteria cultures. Recent studies comparing ability of this system with biochemical analyses showed that MALDITOF appeared to be a rapid and accurate mode of viridans group streptococci identification [33]. This method yielded the correct identification of *S. gordonii* in our patients as confirmed by 16S rRNA gene sequence analysis.

Treatment of septic arthritis differs depending on the joint: native vs prosthetic joint. Prompt treatment with antibiotics together with removal of any purulent material is the mainstay of treatment for septic arthritis.

There is little evidence on which to base the choice and duration of antibiotic treatment, and we found no prospective randomised, controlled trials. One systematic review and meta-analysis of antibiotic treatment for joint infections showed no advantage on clinical or bacteriological efficacy of one antibiotic regimen over another [34]. Currently, the choice of antibiotic is based on the likelihood of the organism involved modified by results of Gram-staining and culture.

Removal of purulent materiel can be achieved either surgically or through closed needle aspiration. There is controversy over which mode of drainage should be used. Recently, Butt et al. found that 77% of rheumatologists and 66% of orthopaedic surgeons (OS) in the UK recommended surgical joint drainage; 22% and 27%, respectively, recommended repeated closed needle aspiration as their chosen method of joint drainage [35]. However, Ravindran et al., in a recent study comparing medical treatment (serial closed-needle aspiration) and surgical treatment (arthroscopy/arthrotomy combined with joint washout), showed that for native joint SA, surgical treatment was not superior to medical treatment and, therefore, highlighted the need for careful case selection for surgical intervention [36].

Antimicrobial therapy without implant removal in streptococcal prosthetic joint infections has been reported to be successful in 45 of 63 cases (71%) [37]. Everts et al., in their study on PJI due to *Streptococcus* treated with antimicrobial therapy and retention of implant, found a success rate of 94% [37]. *Streptococcus viridans* PJI treated with primary antibiotic and implant retention showed success in 8 of 10 cases (80%) [37]. However, some criteria must be respected: stable implant, acute infection with symptom duration of less than 3 weeks and the patient should tolerate long-term antimicrobial therapy. In our second case, we decided to perform a two-stages surgery because symptoms were present for 4 weeks and X-ray showed suspicion of implant loosening.

## Conclusions

We can conclude that, although implication of *S. gordonii* as aetiologic agent of subacute endocarditis is well known, this organism is a rare cause of septic arthritis. In this case, the exclusion of associated endocarditis is warranted. The prognosis of septic arthritis caused by *S. gordonii* appears to be favourable when a rapid approach including drainage of the joint, removal of any prosthesis and antibiotic therapy is undertaken.

## Consent

Written informed consent was obtained from the two patients for publication of these cases reports . A copy of the written consent is available for review by the editor-in-chief of this journal.

## Competing interests

No conflics of interest to declare.

## Authors’ contributions

YJC: Clinical management of the cases and manuscript redaction. BL, DW, BV: relecture of the manuscript. SJ: english correction and redaction of manuscript. OC: surgical management and relecture of manuscript. HR: microbiological management and redaction of manuscript. All authors read and approved the final manuscript.

## Pre-publication history

The pre-publication history for this paper can be accessed here:

http://www.biomedcentral.com/1471-2334/12/215/prepub
